# Intraneural Hemangioma: Case Report of a Rare Tibial Nerve Lesion

**DOI:** 10.7759/cureus.3784

**Published:** 2018-12-27

**Authors:** Steven Kwong, Leanne L Seeger, Kambiz Motamedi, Scott D Nelson, Behrad Golshani

**Affiliations:** 1 Radiology, David Geffen School of Medicine at University of California Los Angeles, Los Angeles, USA; 2 Pathology, David Geffen School of Medicine at University of California Los Angeles, Los Angeles, USA

**Keywords:** intraneural hemangioma, intraneural capillary hemangioma, tibial nerve, peripheral nerve, mesodermal lesion, benign soft tissue lesion, popliteal fossa

## Abstract

An intraneural hemangioma is a rare, benign mesodermal lesion. We present a case of a three-year-old female with the inability to straighten her right knee and fullness over the right popliteal fossa for one year. Magnetic resonance imaging (MRI) demonstrated a T2 hyperintense lesion of the popliteal fossa, within the tibial nerve. The patient underwent an uncomplicated right knee excisional biopsy, which confirmed the diagnosis of an intraneural hemangioma. Although rare, an intraneural hemangioma should be considered in the differential diagnosis of a soft tissue lesion located in the expected course of a peripheral nerve.

## Introduction

An intraneural hemangioma is a rare, benign mesodermal lesion [1-4]. The first case was reported by Sommer in 1922 [5]. Since then, several other case studies have been reported involving various peripheral nerves [2]. There are currently no established treatment guidelines, although surgical excision with nerve graft, when possible, is curative. Intraoperatively, these lesions are adherent to the nerve fibers and may arise from either the nerve or nerve sheath. There is a risk of recurrence with subtotal resection [1,3]. We report a case of a three-year-old female with an intraneural hemangioma of the tibial nerve.

## Case presentation

A three-year-old female presented with the inability to straighten her right knee and fullness over the right popliteal fossa for one year. There was no history of trauma or other pertinent past medical history. The patient denied significant activity-related or night pain, fevers, chills, night sweats, or weight loss. She had no reported sensory or motor nerve deficit. 

Upon presentation, vital signs and laboratory tests were within normal limits. On physical exam, the right knee was held in flexed position at rest with visible fullness in the popliteal fossa. The right lower extremity also appeared larger than the contralateral side. The patient’s gait revealed decreased right stride length and the inability to extend her right knee.

**Figure 1:**
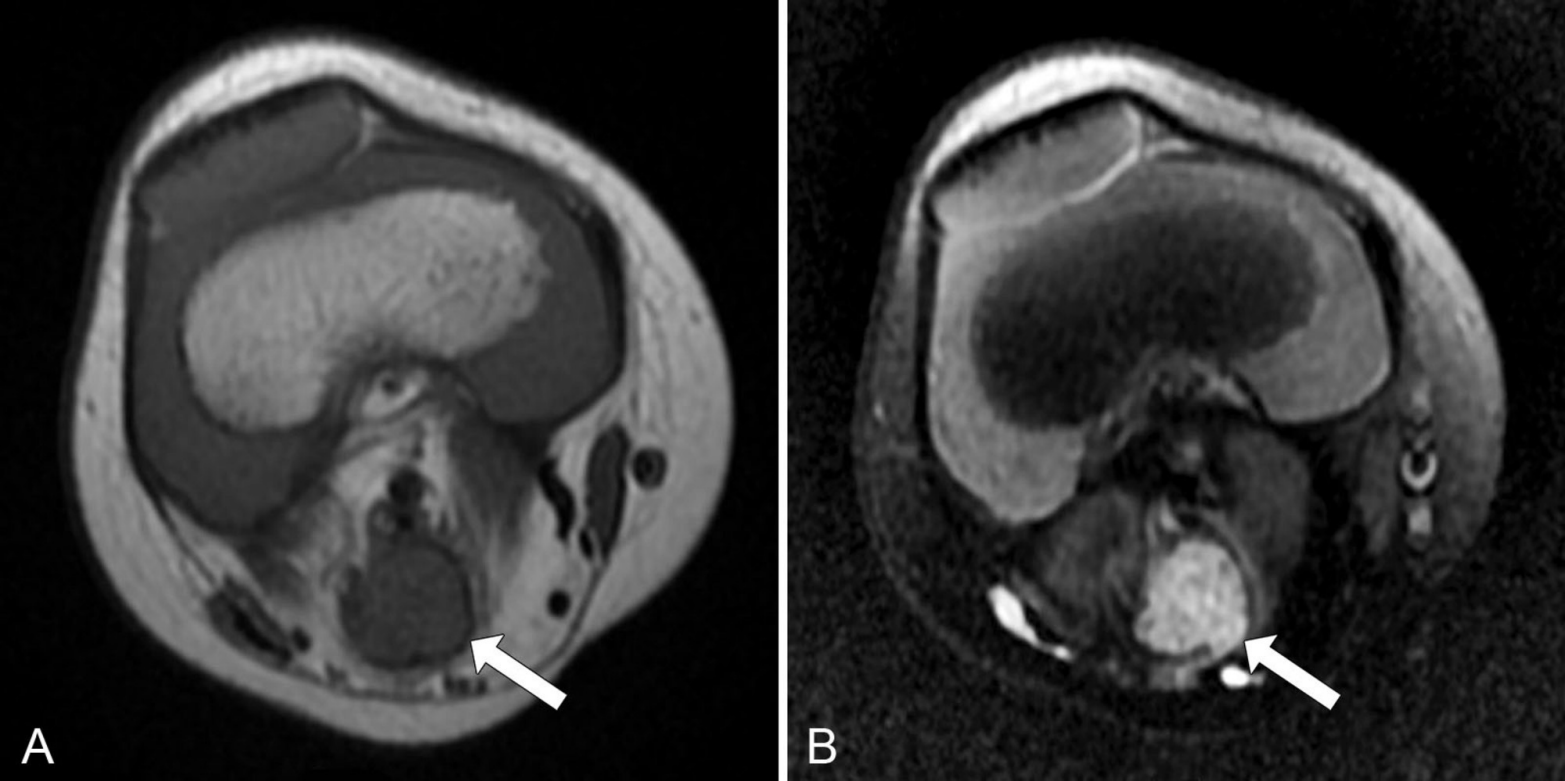
Lobulated lesion in the popliteal fossa (A) Axial T1-weighted image demonstrates a lesion in the popliteal fossa (arrow) isointense to surrounding muscle. (B) Axial proton density fat-saturated image shows that the same lesion (arrow) is heterogeneously hyperintense.

Magnetic resonance imaging (MRI) of the right knee demonstrated a 1.8 x 1.2 x 1.3 cm (craniocaudal x transverse x anteroposterior (AP)) lobulated lesion within the popliteal fossa in direct continuity with the tibial nerve. The lesion was T1 isointense to muscle (Figure 1A) and was heterogeneously hyperintense on proton density fat-saturated (Figure 1B) sequences. Gadolinium was not administered during the study.

**Figure 2:**
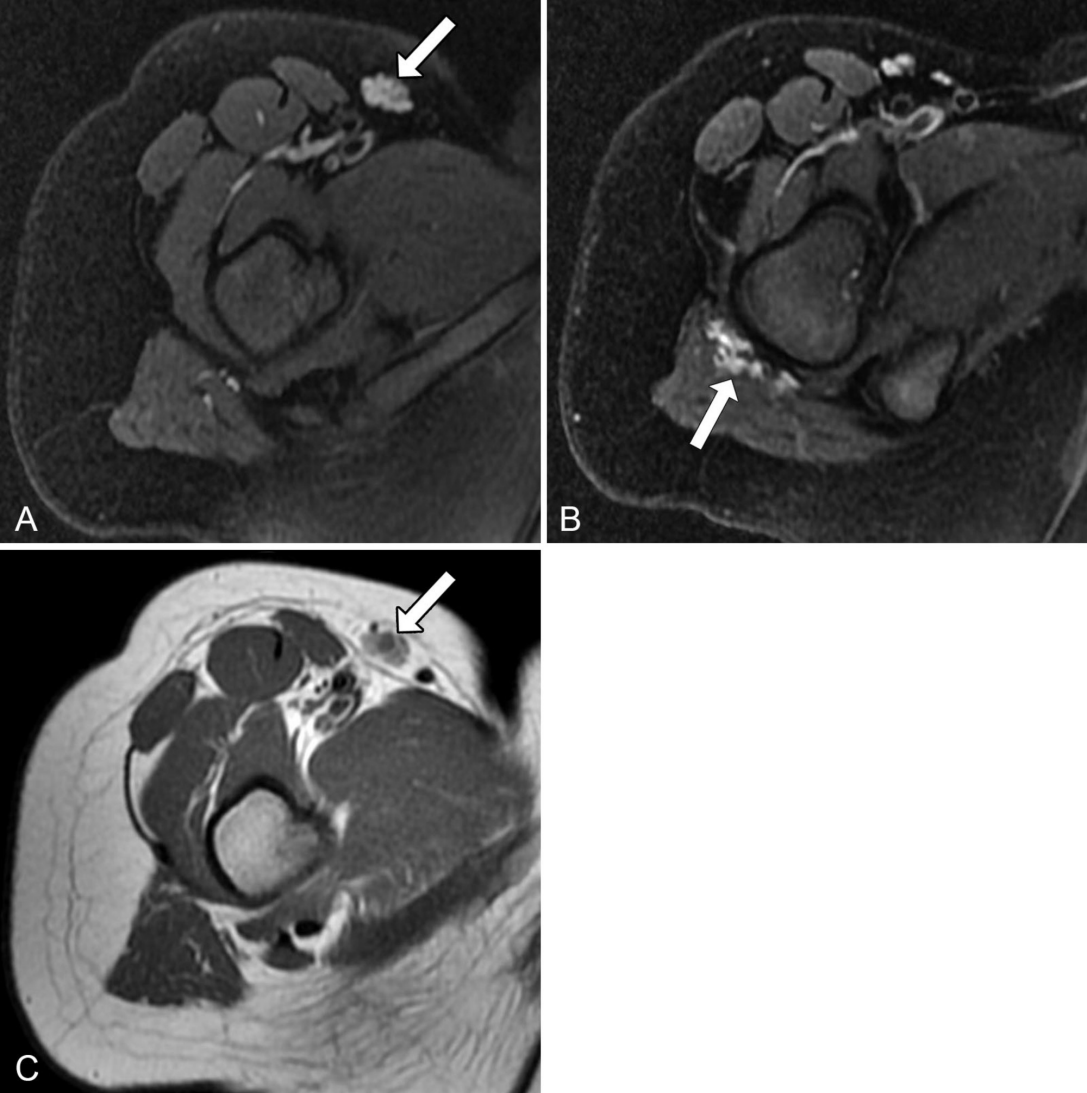
Similar-appearing lesions in the right inguinal subcutaneous soft tissues and gluteus maximus muscle belly (A, B) Axial proton density fat-saturated images demonstrate additional lesions (arrows) in the right inguinal subcutaneous soft tissues and gluteus maximus muscle belly with similar signal characteristics compared to the lesion in the popliteal fossa. However, (C) T1-weighted axial image demonstrates that, unlike the lesion in the popliteal fossa, the lesion in the right inguinal subcutaneous soft tissues (arrow) has a high intrinsic T1 signal.

More proximally, there were additional lesions within the right inguinal subcutaneous soft tissues (Figure 2A) and the gluteus maximus muscle belly (Figure 2B), which demonstrated signal characteristics similar to the lesion within the popliteal fossa with the exception of high intrinsic T1 signal (Figure 2C).

**Figure 3:**
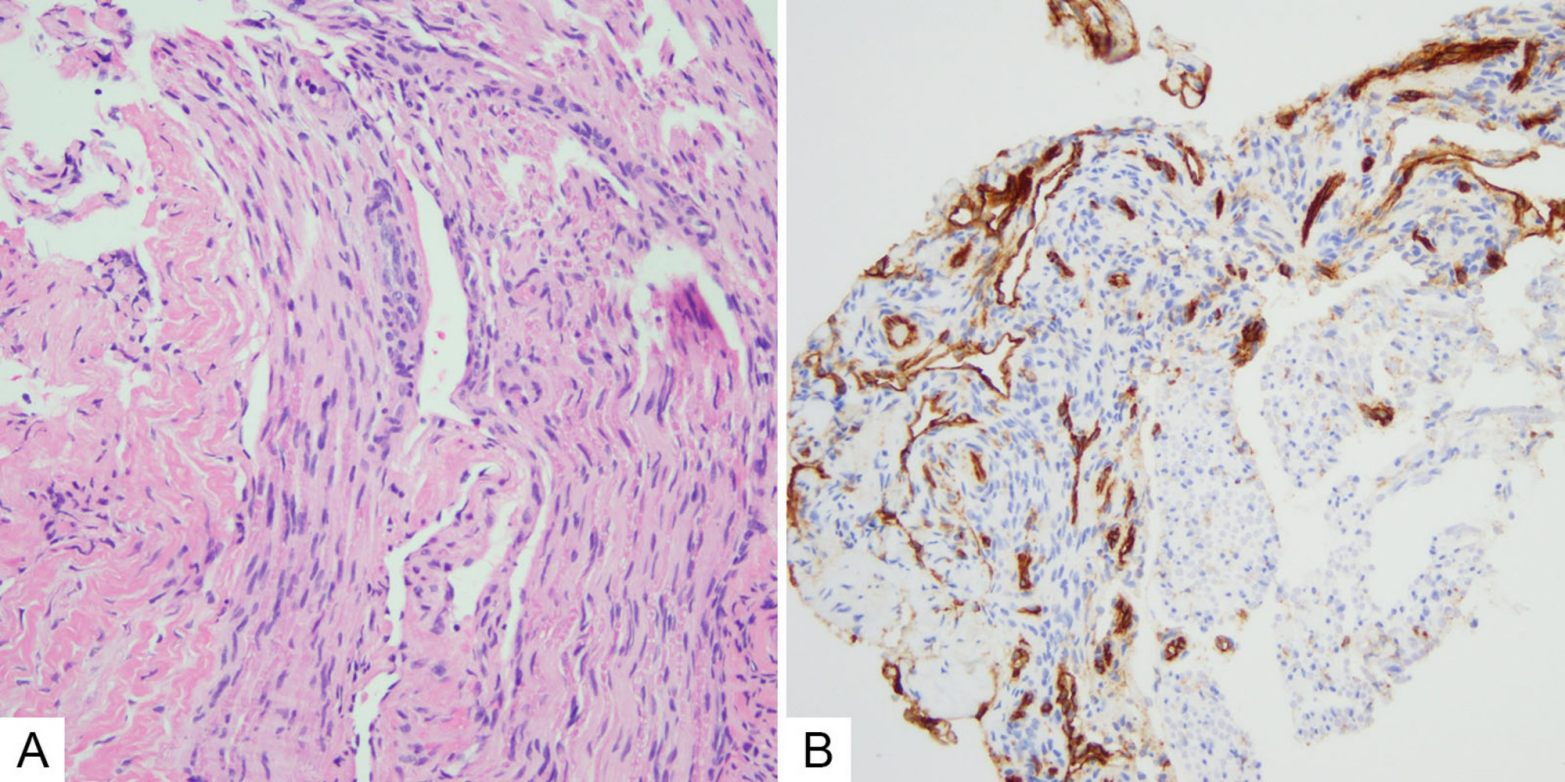
Histologic images consistent with an intraneural hemangioma (A) Hematoxylin and eosin demonstrated nerve tissue with intervening vascular spaces of varying sizes lined by bland epithelium. (B) CD31 immunohistochemistry positively stained the epithelial cells.

The patient underwent an uncomplicated right knee mass excisional biopsy. Intraoperatively, the mass was noted to be adherent to the nerve with dark coloration and as much of the lesion as possible was removed without putting the tibial nerve at risk. A hematoxylin and eosin (H and E) stain demonstrated nerve tissue with intervening vascular spaces of varying sizes lined by bland epithelium (Figure 3A), and CD31 immunohistochemistry positively stained the epithelial cells (Figure 3B).

Postoperatively, the patient did not have any major complaints or side effects. She continues to be under close surveillance by the orthopedic oncology department.

## Discussion

Intraneural hemangiomas have been described involving several different peripheral nerves; however, the involvement of the median nerve has been the most commonly reported [2-3]. Patients generally present with sensory or motor deficits related to the involved nerve [1-3]. In most, a palpable mass was reported along the expected course of a nerve. To the best of our knowledge, our case is the fourth reported case in the literature of an intraneural hemangioma involving the tibial nerve [2].

The differential diagnoses of these lesions include schwannomas, neurofibromas, lipofibromas, hamartomas, and malignant peripheral nerve sheath tumors [1,3].

On ultrasound, intraneural hemangiomas appear as well-defined hypoechoic structures with posterior acoustic enhancement. Although ultrasound was not used to evaluate our patient, it may be useful for providing real-time, dynamic information on the tumor’s vascularity and relationship to the surrounding soft tissue structures [1-3].

MRI provides preoperative information regarding the anatomic location, size, and relationship of the tumor to the nerve and surrounding structures. These tumors classically show areas that are hyperintense on T1-weighted and T2-weighted fat-suppressed sequences, although the T1 appearance may be variable with the flow voids, feeding veins, and draining vessels occasionally seen. There is, classically, gadolinium enhancement on post-contrast images [1-3].

In our case, the multiplicity of the lesions helped narrow the differential diagnosis, with the more proximal lesions demonstrating signal characteristics suggestive of a hemangioma. A possible explanation of the lack of intrinsic T1 signal of the lesion of interest within the popliteal fossa may be due to its size and subsequent internal architectural distortion.

Conservative management of these lesions is often unsuccessful. Total surgical excision along with nerve graft, when possible, is curative. However, this may not be feasible depending on the involved nerve and relationship with the surrounding structures. In these cases, partial resection may relieve symptoms, although recurrence is possible [1-4]

Chatillion et al. reported the first case of radiotherapy for the treatment of intraneural hemangioma. His study demonstrated symptomatic relief and radiologic shrinkage in size of an intraneural hemangioma involving the inferior trunk of the brachial plexus [6]. Additional case reports have reported symptom relief with radiotherapy for unresectable hemangiomas [7].

## Conclusions

Intraneural hemangiomas, while rare, should be kept in the differential for intraneural lesions. Presenting symptoms may vary and the multiplicity of hemangiomas elsewhere may help narrow the differential.
